# Dynamic change patterns of the human gut microbiota—fluctuation, loss-acquisition, and turnover—and their underlying causes

**DOI:** 10.1093/ismeco/ycag046

**Published:** 2026-02-28

**Authors:** Wen Zhang, Na Han, Tingting Zhang, Yujun Qiang, Xianhui Peng, Xiuwen Li, Biao Kan

**Affiliations:** National Key Laboratory of Intelligent Tracking and Forecasting for Infectious Diseases, National Institute for Communicable Disease Control and Prevention, Chinese Center for Disease Control and Prevention, Beijing 102206, China; National Key Laboratory of Intelligent Tracking and Forecasting for Infectious Diseases, National Institute for Communicable Disease Control and Prevention, Chinese Center for Disease Control and Prevention, Beijing 102206, China; National Key Laboratory of Intelligent Tracking and Forecasting for Infectious Diseases, National Institute for Communicable Disease Control and Prevention, Chinese Center for Disease Control and Prevention, Beijing 102206, China; National Key Laboratory of Intelligent Tracking and Forecasting for Infectious Diseases, National Institute for Communicable Disease Control and Prevention, Chinese Center for Disease Control and Prevention, Beijing 102206, China; National Key Laboratory of Intelligent Tracking and Forecasting for Infectious Diseases, National Institute for Communicable Disease Control and Prevention, Chinese Center for Disease Control and Prevention, Beijing 102206, China; National Key Laboratory of Intelligent Tracking and Forecasting for Infectious Diseases, National Institute for Communicable Disease Control and Prevention, Chinese Center for Disease Control and Prevention, Beijing 102206, China; National Key Laboratory of Intelligent Tracking and Forecasting for Infectious Diseases, National Institute for Communicable Disease Control and Prevention, Chinese Center for Disease Control and Prevention, Beijing 102206, China

**Keywords:** gut microbiota, dynamic changes, strain turnover, host genetic factors, time

## Abstract

The temporal dynamics of the gut microbiome are critical to human health, yet their patterns and underlying drivers remain poorly characterized at a monthly resolution and strain level. This knowledge gap limits the development of targeted microbiome interventions. Here, we integrate longitudinal analyses across three human cohorts—a cross-sectional cohort (*n* = 190), an intensive 52-month time series (*n* = 7), and a paired 6-month cohort (*n* = 43)—together with a humanized mouse model under antibiotic perturbation. Using shotgun metagenomics (516 samples), we resolve microbial dynamics at species and strain resolution. We identify three distinct modes of temporal variation: relative abundance fluctuations, species loss-acquisition events, and strain turnover. Strain turnover contributes substantially to the dynamic reservoir of functional genes, including those associated with virulence and antibiotic resistance. These dynamics are influenced by antibiotic exposure and microbial interspecies interactions. Our work provides a month-scale atlas of gut microbiome variation, revealing widespread transient colonization and strain-level plasticity, thereby offering a refined framework for understanding microbiome stability and personalized microbial ecology.

## Graphical Abstract

**Graphical Abstract:**
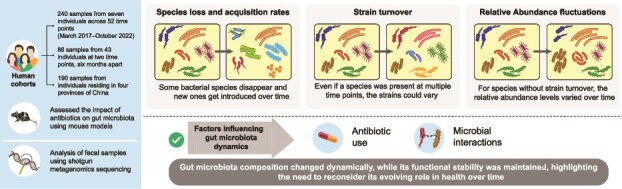

## Introduction

The gut microbiome is a dynamic ecosystem intricately linked to human health and disease, with specific microbial signatures serving as potential biomarkers for various conditions [1–4]. Its composition is shaped by factors such as age, diet, environment, and host genetics over the lifespan [5–8]. Although daily lifestyle can drive rapid community shifts [9], advances in metagenomic profiling have unveiled another layer of complexity: strain-level dynamics that govern microbial persistence and turnover [10–12]. Although strain-resolved analyses have revealed patterns of long-term colonization and adaptation [13, 14], a systematic understanding of temporal strain dynamics across healthy populations—particularly at high (e.g. monthly) resolution—remains limited.

To address this gap, we established the Chinese Healthy Gut Microbiome Project (CMP), a longitudinal initiative involving monthly stool sampling from healthy individuals [8, 15]. Here, we conduct a deep, strain-resolved analysis of gut microbiome dynamics using shotgun metagenomics and integrate survey data and controlled animal experiments to identify key drivers of microbial change. Building on our earlier observations of species-specific temporal patterns in taxa such as *Escherichia coli* and *Segatella* [16–18], this study aims to uncover universal principles that govern the stability and turnover of the gut microbiome across time and individuals.

## Materials and methods

### Study design and sample collection protocol for the Chinese Healthy Gut Microbiome Project cohort

**Figure 1:**
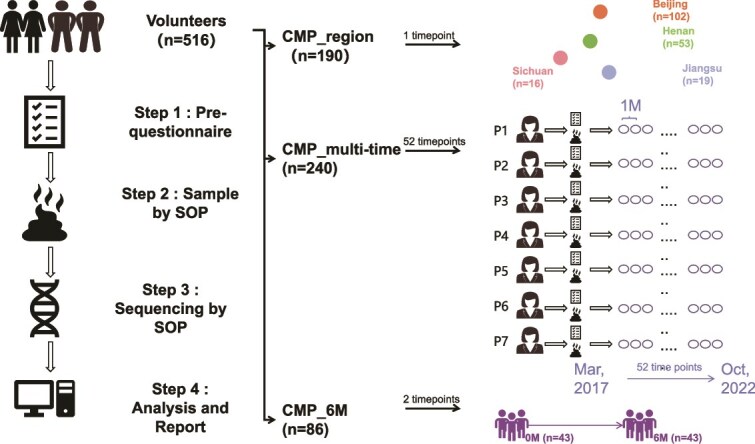
Overview of the CMP study design. Schematic illustrating the sampling framework, cohort structure, and analytical workflow for the three subprojects: CMP_region (cross-sectional), CMP_multi-time (longitudinal monthly sampling), and CMP_6M (paired 6-month sampling).

The present study used samples from three subprojects of the CMP (Fig. 1).

1. CMP_region: Fecal samples were collected from 190 individuals across four provinces of China: Beijing, Jiangsu, Henan, and Sichuan. These regions represent the northern, southern, central inland, and western regions of China, respectively [15].
2. CMP_multi-time: This subproject included 240 fecal samples obtained from seven healthy Chinese individuals across 52 time points between March 2017 and October 2022.
3. CMP_6M: A total of 86 fecal samples were collected from 43 healthy volunteers from the same regions at two time points: 0 and 6 months.

All three subprojects adhered to the following methodology: samples were collected from healthy Chinese individuals who provided written informed consent before enrollment in the study. The eligibility criteria for participation were as follows: (i) age 18–70 years at the time of enrollment; (ii) body mass index (BMI) between 16 and 30; (iii) participants must be healthy and willing, as well as able to provide stool specimens and complete a detailed monthly questionnaire; (iv) blood pressure <140/90 mmHg and postprandial blood glucose levels <11.1 mmol/l; and (v) no history of cancer, tuberculosis, surgical illnesses, or 40 other conditions in the participants and their immediate family members. At each sampling time point, the volunteers were asked to complete a questionnaire regarding their medication use and medical history in the month leading up to the sample collection. Height, weight, blood pressure, and blood glucose levels were measured on-site. All fecal samples were collected within 24 h after finishing the pretest and prequestionnaire. The feces were collected using a disposable bedpan provided to the volunteers, rather than from a flush toilet to avoid contamination by water in the toilet bowl.

### Mouse experiments

Gnotobiotic C57BL/6 mice (8 weeks old; male) were obtained from Shenzhen Gnotobio Biotechnology Co., Ltd. (Shenzhen, China) to establish murine models using previously described methods [19, 20]. To create a humanized gut microbiota model, we performed oral gavage three times with 200 μl of fecal microbial suspension. After 7 days of model establishment, antibiotic intervention was conducted on the first group of mice (*n* = 10) using ceftriaxone (6 mg per mouse, dissolved in 200 μl Phosphate Buffered Saline (PBS) and administered via oral gavage). The control group (*n* = 10) only received 200 μl of PBS via oral gavage. Fecal samples were collected from each mouse at three time points: before gavage (Day 0) and on Days 1 and 4 immediately after gavage. DNA extraction, library preparation, sequencing, and bioinformatics analysis were performed using standardized protocols as described for human samples.

### DNA extraction, sequencing, and quality control analysis

Fresh stool samples were transported to the laboratory within 2 h of collection using insulated containers maintained at 4°C. Upon arrival, 200 ± 20 mg aliquots were immediately processed for DNA extraction with the QIAamp Fast DNA Stool Mini Kit (Qiagen, Cat# 51604, Hilden, Germany), strictly adhering to the manufacturer's protocol with critical enhancements: each aliquot received 1 ml of InhibitEX buffer followed by vigorous homogenization via vortex mixing at maximum speed for 1 min until complete sample dissolution. The homogenate was centrifuged at 20 000× g for 1 min at 4°C (Eppendorf 5424 R); If particulate matter persisted in the supernatant, centrifugation was repeated under identical conditions. A 600 μl volume of clarified supernatant was then transferred to sterile microcentrifuge tubes for subsequent extraction steps. All extractions were completed within 24 h of sample receipt. Extracted DNA underwent stringent quantification using a Qubit 4.0 Fluorometer with dsDNA HS Assay Kit (Thermo Fisher, Q32854), and only samples exceeding 10 ng/μl concentration were proceeded to downstream analysis.

The shotgun metagenomics library was constructed using the TruePrep DNA Library Prep Kit (Illumina) and sequenced using an Illumina HiSeq platform to generate 150 bp paired-end reads. Low-quality reads were removed using FastQC and Fastp [21]. Bases with quality scores lower than Q20 from the 3' end of the reads were trimmed, retaining only the reads with a minimum length of 100 bp as high-quality reads. Each sample yielded more than 15 million high-quality reads, with over 80% of the bases scoring above Q30. Subsequently, human sequences were removed using Kraken2 [22]. The total amount of high-quality sequencing data analyzed reached 5 terabytes in this study, with an average of 32 million high-quality reads per sample. All sequencing data in the project, as well as the metadata (Table S1), have been deposited in the NCBI Sequence Read Archive (SRA) database (SAMN44930011-SAMN44930200 for CMP_region; SAMN41553299-SAMN41553537 and SAMN47078108 for CMP_multi-time; SAMN47078022-SAMN47078107 for CMP_6M).

### Species and strain analysis for samples sequenced via the shotgun metagenomics method

The analysis pipeline used in the present study is available on GitHub (https://github.com/zhangwencdc/GutTime).

For qualified samples, the relative abundance of bacterial species was estimated using MetaPhlAn4 [12, 23]. Only species with a relative abundance ≥0.01% were retained for subsequent analysis. Species loss, acquisition, and strain turnover rates were calculated using a custom Perl script. Alpha and beta diversity metrics were computed across all samples using the microeco [24] and MicrobiotaProcess [25] R packages, respectively.

Metagenomic reads were assembled using individual sample-specific SPAdes v3.13.0 with --meta flag [26]. The resulting contigs were binned into metagenome-assembled genomes (MAGs) using MetaBat v2.12.1 [27]. MAG quality was assessed with CheckM2 [28]; only MAGs meeting high-quality thresholds (completeness >90%, contamination <5%) were retained. Taxonomy was assigned via GTDB-Tk v2.4.1 [29] against GTDB r207, retaining only classifications with ≥70% alignment breadth and >95% alignment identity.

For strain-level comparisons, pairwise average nucleotide identity (ANI) was calculated with FastANI [30] (minimum fragment length 1000 bp). Following established cut-offs, MAG pairs with ANI ≥ 99.95% were considered the shared strain, those with ANI < 99.5% were classified as distinct strains, and intermediate values (99.5% ≤ ANI < 99.95%) were treated as ambiguous and excluded from strain-turnover calculations [12, 31]. For phylogenetic visualization, MAGs belonging to the same species were compared using ANIclustermap (https://github.com/moshi4/ANIclustermap) and the resulting trees were annotated with iTOL [32].

A co-occurrence network of the gut microbiota was constructed using ggplot2 [33]. Spearman correlation analysis was performed between bacterial species; only correlations with coefficients |*r*| > 0.6 and *P*-values <0.001 were retained.

### Detection of potential virulence factors and antibiotic resistance genes

Potential virulence factors (VFs) were identified using the Bowtie2 [34] method based on the Virulent Factors of Pathogen Database (VFDB) [35]. The aligned length for each VF was calculated, and only those with a coverage percentage (coverage% = aligned length/gene size) >90% were considered positive results. The same pipeline was applied for detecting antibiotic resistance genes (ARGs) utilizing the ResFinder database (https://cge.cbs.dtu.dk/services/ResFinder/). We used the HumanN [36] tool to analyze the gene families present in the samples and assess the coverage of various pathways.

### Statistical analyses

Statistical significance was assessed using Wilcoxon rank-sum tests with false discovery rate (FDR) correction (Benjamini–Hochberg procedure). Significance was defined as FDR-adjusted *P* <0 .05. Data were visualized with ggplot2 v3.4.2 [33].

## Results

To compare the effects of temporal features on the human gut microbiome, we established a comprehensive 5-year multi-time point cohort (CMP_multi-time cohort, Fig. 1) and collected fecal samples at 52 time points from seven individuals between March 2017 and October 2022, each corresponding to a month apart (Fig. 1). At each sampling time, the seven volunteers were assessed. Height, weight, blood pressure, and blood glucose levels were measured on-site every month for every volunteer, and a detailed report on their living habits (including smoking, physical exercise, fruit and alcoholic drink consumption) as potential factors affecting the gut microbiome was compiled based on questionnaires. After excluding samples that failed during sampling, DNA extraction, or sequencing, a total of 240 fecal samples were successfully collected and sequenced (Fig. 1), with an average of 32 million high-quality reads per sample. We identified 593 bacterial species from this CMP_multi-time cohort study dataset using Metaphlan4 [12, 23].

### Fluctuations in the gut microbiota

**Figure 2:**
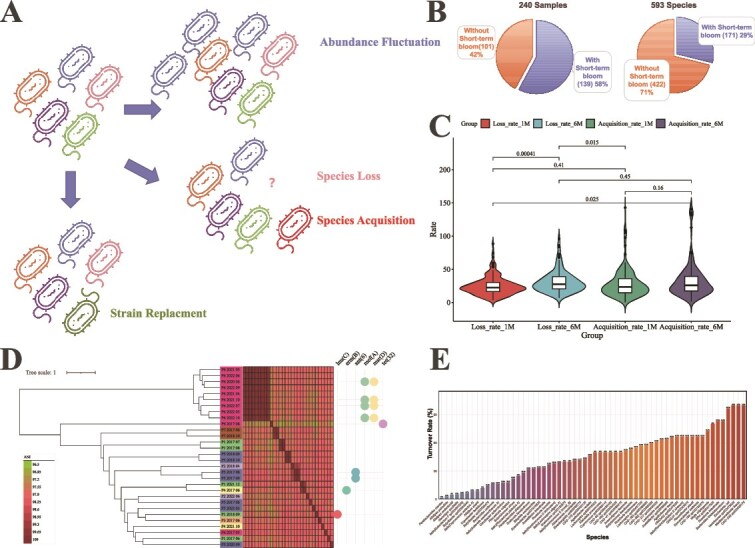
Temporal dynamics of the gut microbiome at the species and strain levels. (A) Conceptual diagram of the three observed dynamic patterns: short-term bloom, species loss/acquisition, and strain turnover. (B) Proportion of samples (left) and species (right) that exhibited short-term bloom events. (C) Violin plots showing species loss rates (left two) and acquisition rates (right two) over 1- (1M) and 6-month (6M) intervals. (D) Phylogenetic tree of 30 high-quality MAGs of *R. inulinivorans*, with associated heatmap depicting pairwise ANI. Right circles indicate the presence of ARGs in corresponding strains. (E) Strain turnover rates across 60 bacterial species with recoverable MAGs.

Consistent with prior 16S rRNA gene sequencing-based findings [8, 37], we observed variations in species relative abundance. Although samples from the same individual were more similar than those from different individuals (Supplementary Fig. 1), fluctuations occurred over time. A total of 139 of the 240 samples (see Supplementary Fig. 1) exhibited a “short-term bloom”, defined as a greater than five-fold change in the relative abundance of at least one species compared to the previous time point. Such blooms have been previously reported for specific taxa like *Akkermansia, Segatella,* and *E. coli* [16–18]. Our analysis revealed that 171 of the 593 species underwent at least one short-term bloom (Fig. 2B), indicating this phenomenon is widespread.

### Transient acquisition or loss of gut microbiota bacteria at the species level

Beyond relative abundance fluctuation, the gut microbiome was also proven to be with compositional change, characterized by the loss and acquisition of species [38]. In this study, we quantified these rates by pairwise comparison of samples from consecutive time points across the 52 time points. The species loss rate—defined as the proportion of species present in an initial sample but absent in a subsequent sample from the same individual—was 24.85% (SD = 13.98%) over 1-month intervals. This rate increased to 31.37% (SD = 16.73%) over 6-month intervals. Concurrently, the species acquisition rate (proportion of newly detected species) was 28.43% (SD = 21.41%) and 33.00% (SD = 26.56%) over 1- and 6-month intervals, respectively (Fig. 2A–C). These results confirm that the gut microbiome undergoes continuous restructuring over short timescales, with more pronounced changes over longer periods.

### Strain turnover in gut microbiota

In this study, we further identified a third mode of dynamics: strain turnover, wherein a persistent species is represented by genetically distinct strains at different time points. For example, *Roseburia inulinivorans* was detected in 184 samples (76.67%). From these samples, we recovered 30 high-quality MAGs. Phylogenetic analysis revealed that strains from the same individual were often distinct; for example, MAGs from participant P6 in May 2017 and August 2017 shared only 97.46% ANI (Fig. 2D). This pattern aligns with our prior observations for *E. coli* [16, 17]. Extending this analysis to all 593 species identified in the CMP_multi-time cohort, we obtained high-quality MAGs for 204 species, and among these, 60 species (29.4%) exhibited evidence of strain turnover during the study period (Fig. 2E). At the genome-pair level for 204 species with high-quality MAGs, 23.8% (3606 out of 15,164) of within-individual high-quality MAG comparisons were classified as distinct strains (ANI < 99.5%). The strain turnover rate was 1.4% (24/1728) for samples 1 month apart, increasing to 2.2% (22/986) for samples 6 months apart.

To validate the prevalence of strain turnover in a larger population, we analyzed a separate cohort (CMP_6M) comprising 43 individuals sampled at 0 and 6 months (Fig. 1). From 182 species with high-quality MAGs, 8 (4.4%) showed evidence of strain turnover. At the genome-pair level, 6.1% (18 out of 296) of within-individual comparisons involved distinct strains, corroborating the phenomenon observed in the CMP_multi-time cohort.

### Functional implications of microbial dynamics

**Figure 3:**
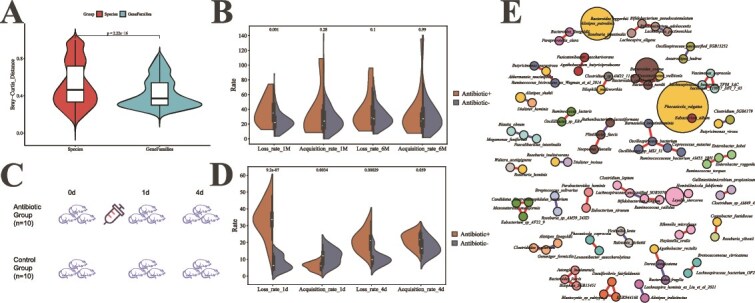
Drivers and functional correlates of gut microbiome dynamics. (A) Comparison of community dissimilarity (Bray–Curtis distance) based on species abundance (left) versus gene family profiles (right). (B) Effect of antibiotic exposure on species loss and acquisition rates over 1-month and 6-month intervals in the CMP_multi-time cohort. (C) Schematic of the humanized microbiota mouse experiment, including antibiotic-treated and control groups. (D) Loss and acquisition rates in mice at 1 day and 4 days after antibiotic treatment. (E) Co-occurrence network of bacterial species, edges represent significant Spearman correlations (|*r*| > 0.6, *P* <0 .001) with red and blue lines indicating positive and negative correlations, respectively. Node size corresponds to mean relative abundance across all samples; yellow nodes denote species that underwent strain turnover in the CMP_multi-time cohort.

We next assessed whether this microbial taxonomic dynamics translated to functional instability. Analysis of 240 longitudinal samples (CMP_multi-time cohort) with HUMAnN3 [36] revealed 743,917 microbial gene families, of which 205,374 (27.6%) were transiently detected (present in only one sample), indicating considerable functional flux. However, pairwise Bray–Curtis dissimilarity was significantly lower for functional profiles than for taxonomic profiles (*P* <0 .01, Wilcoxon test; Fig. 3A), suggesting core metabolic functions are more stable than community composition.

The dynamic patterns of new species and strain turnover, however, had specific functional consequences: the fluctuating carriage of bacterial potential VFs and ARGs. Screening against the VFDB database identified 2,512 putative VFs in 240 longitudinal samples (CMP_multi-time cohort), all of which appeared transiently in a subset of samples (Supplemental Fig. 2). Similarly, we identified 330 ARGs across these samples, 302 (91.52%) of which appeared only transiently in a subset of samples (Supplemental Fig. 3), indicating a highly dynamic resistome.

Genome-level analysis directly linked strain turnover to changes in resistance profiles. For instance, comparing *R. inulinivorans* MAGs from participant P6 (August 2017 vs. June 2020) revealed not only low ANI (96.79%) but also distinct ARG complements: the earlier strain carried a tetracycline resistance gene *tet(32)*, and the later strain carried macrolide resistance genes *mef(A)* and *msr(D)* (Fig. 2E).

### Drivers of gut microbiota dynamics

Building on our understanding that dynamic changes occur in the gut microbiota of healthy individuals, we further explored the potential causes underlying these changes.

Antibiotic usage is a well-known disruptor of gut microbial communities [39]. Our longitudinal data confirm its role and delineate the temporal scope of its effects. Analysis of self-reported antibiotic use across the 5-year study (CMP_multi-time cohort) revealed that the species loss rate over 1-month intervals was significantly elevated following antibiotic exposure compared to nonexposure periods (Fig. 3B). However, this effect was not sustained: no significant difference in loss rate was observed between the antibiotic and control groups over 6-month intervals. Furthermore, antibiotic use showed no significant impact on species acquisition rates at either the 1- or 6-month scale (Fig. 3B). These results indicate that antibiotics primarily induce short-term species loss without altering longer-term dynamics.

Controlled animal experiments corroborated this time-dependent effect. In a humanized gut microbiota mouse model (Fig. 3C), both species loss and acquisition rates were significantly higher one day post-antibiotic treatment than in controls. By day four post-antibiotic, the acquisition rate difference was no longer significant, and although the loss rate remained elevated, the disparity had diminished (Fig. 3D). This pattern reinforces the transient nature of antibiotic-driven disruption.

In the humanized gut microbiota mouse model, which is under gnotobiotic conditions, no strain turnover was identified in either antibiotic-treated or control samples, suggesting that dietary or environmental microbial exposure is a candidate source for strain turnover.

Beyond external perturbations, intrinsic ecological interactions within the gut community appeared to influence dynamics. Co-occurrence network analysis in the CMP_multi-time cohort identified 90 significant pairwise correlations (47 positive, 16 negative) between species relative abundances (Fig. 3E). A total of 12 of the species involved in these interactions were also among those observed to undergo strain turnover, hinting that interspecific relationships may be linked to strain-level instability.

Finally, we tested the hypothesis that a species' prevalence in a population may be related to its stability within an individual host. We reasoned that highly prevalent species might possess traits conferring colonization resilience, leading to lower turnover rates. Species prevalence was calculated from the cross-sectional CMP_region cohort (*n* = 190, Fig. 1) and compared to their strain turnover rates derived from the longitudinal CMP_multi-time cohort. No significant correlation was observed (Supplemental Fig. 4). An example is *R. inulinivorans*, which exhibited high population prevalence (87.76%) in the CMP_region cohort yet also a high strain turnover rate within individuals over time in the CMP_multi-time cohort (Fig. 2E). This finding suggests that commonality in a population is not a reliable proxy for stable colonization within a personal microbiome.

## Discussion

Our longitudinal, strain-resolved analysis reveals that the healthy gut microbiome is characterized by a multilayered dynamism. Beyond the well-documented fluctuations in species relative abundance and species composition, we identify strain turnover as a fundamental and widespread mode of change. This work demonstrates that even in the absence of overt disease, an individual's gut microbiota undergoes continuous remodeling at the strain level, with functional implications for pathogenicity and antibiotic resistance. These findings compel a reevaluation of what constitutes a “stable” gut microbiome and highlight new dimensions of microbial individuality.

### The significance of strain turnover: an underestimated dimension of microbial flux

The ecological stability of the gut microbiome has historically been assessed through the persistence of taxonomic units at the species level. Our data reveal a more profound layer of instability: nearly one-third (60/204) of tracked species with high-quality genomes underwent strain replacement over a 5-year period. This genomic turnover occurs silently within stable species-level profiles, meaning a significant portion of microbial community dynamics is masked by conventional analytical resolution. The strain turnover rate, quantifiable across different time intervals, provides a metric to capture this hidden ecosystem flux. Its prevalence suggests that the bacterial turnover is a routine event, potentially originating from environmental or dietary sources, as hinted by our mouse experiments. Therefore, a complete picture of gut microbiome dynamics must integrate species relative abundance, species acquisition/loss, and strain-level turnover.

### Implications for the host: redefining personal microbial identity

The dynamics of the gut microbiota adds a temporal dimension to the individuality of individuals: each person harbors not only a unique set of species but also a unique pattern of strain-level flux. The ecological relationships that influence dynamics are likely shaped by the host’s unique physiological and immunological landscape. Consequently, an individual’s health or disease state may be linked not merely to the presence of certain species, but to the stability landscape of their constituent strains. This frames the gut microbiome not as a static fingerprint but as a personalized, evolving ecosystem with its own historical contingencies and turnover rules.

### Implications for health assessment: stability as a potential biomarker

The functional consequences of strain turnover, particularly the transient introduction of potential VFs and ARGs, directly link microbial dynamics to health risk. This suggests that microbial instability itself could be a biomarker. In clinical or wellness contexts, assessing gut health may therefore evolve beyond cross-sectional snapshot profiling of species composition. Monitoring strain persistence over time, measuring turnover rates of key taxa, or tracking the flux of risk-associated gene pools could provide more sensitive indicators of ecosystem resilience, dysbiosis, or response to interventions. A “stable” microbiome, in this new view, might be defined not only by the absence of change, but also by the regulated pace and functional neutrality of its strain-level succession.

### Limitations

This study has several methodological limitations. First, our strain-turnover analysis relies on reconstructing high-quality MAGs. Because low-abundance strains frequently yield insufficient sequencing coverage for reliable genome recovery, the strain-turnover rates reported here likely represent a conservative estimate. Second, species-level detection was based on MetaPhlAn4 profiles (threshold ≥0.01% relative abundance). Thus, some observed species-gain and -loss events may reflect methodological detection limits rather than true ecological colonization or extinction. Third, the humanized-mouse experiment employed a standardized fecal inoculum derived from a single healthy donor, and direct longitudinal profiling of the donor’s microbiome was not performed. This design limits the generalizability of the mouse findings to multidonor or personalized microbial contexts. Together, these constraints underscore the need for deeper sequencing, refined bioinformatic pipelines, and multidonor experimental models to fully resolve strain-level dynamics, especially within low-abundance and rare microbial populations.

## Supplementary Material

Supplementary_material_ycag046

## Data Availability

All sequencing data in the project, as well as the metadata (Table S1), have been deposited in the NCBI SRA database (PRJNA1116923, SAMN44930011-SAMN44930200 for CMP_region; SAMN41553299-SAMN41553537 and SAMN47078108 for CMP_multi-time; SAMN4707-8022-SAMN47078107 for CMP_6M). The analysis pipeline used in the present study is available on GitHub (https://github.com/zhangwencdc/GutTime).
