# Supplementary material for: Dynamic change patterns of the human gut microbiota—fluctuation, loss-acquisition, and turnover—and their underlying causes
Source: ISME Commun. 2026 Feb 28;6(1):ycag046. doi: 10.1093/ismeco/ycag046 (PMC13064649; doi:10.1093/ismeco/ycag046)
Supplement: Supplementary_material_ycag046 [file supplementary_material_ycag046.zip › Supplemental Figure and Table legends.docx]

**Supplemental Materials**

**Figure S1. Species abundance profiles and community dissimilarity in longitudinal samples.**

(A–G) Stacked bar plots showing the relative abundance of the top 15 bacterial species in each sample from participants P1 to P7. Remaining species are summarized in grey. Samples marked in red along the x-axis indicate time points where a short‑term bloom was detected.

(H) Violin plots comparing Bray-Curtis distances between samples from the same individual (left) and between different individuals (right).

**Figure S2. Distribution of virulence factor prevalence across individuals.**

Violin plots show the prevalence of potential virulence factors (derived from VFDB) in fecal samples from the seven longitudinally sampled participants (P1-P7).

**Figure S3. Temporal variation of antibiotic resistance genes (ARGs).**

Heatmap depicting the presence/absence of 330 detected ARGs across samples from the seven individuals over time. Rows represent ARGs, columns represent samples.

**Figure S4. Relationship between species prevalence and strain turnover rate.**

Scatter plot illustrating longitudinal strain turnover rates (x‑axis, derived from CMP_multi‑time) against species prevalence (y‑axis, percentage of positive samples in the CMP_region cohort). No significant correlation was observed.

**Table S1. Metadata for all samples included in the study.**

Comprehensive sample‑associated information, including cohort origin, sampling time, participant ID, and questionnaire‑derived lifestyle variables.
