# Supplementary figures and images for: Dynamic change patterns of the human gut microbiota—fluctuation, loss-acquisition, and turnover—and their underlying causes
Source: ISME Commun. 2026 Feb 28;6(1):ycag046. doi: 10.1093/ismeco/ycag046 (PMC13064649; doi:10.1093/ismeco/ycag046)

A

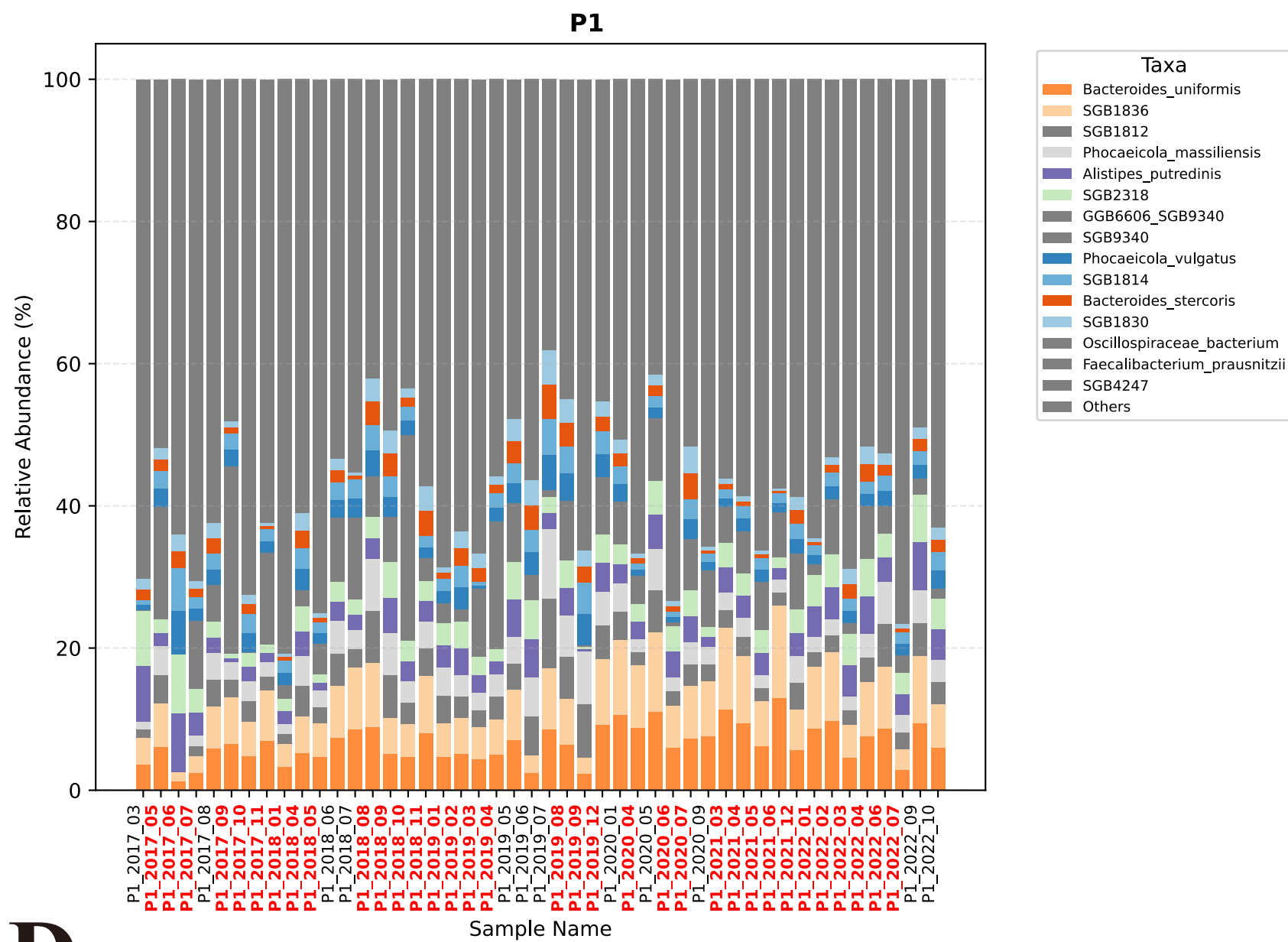

B

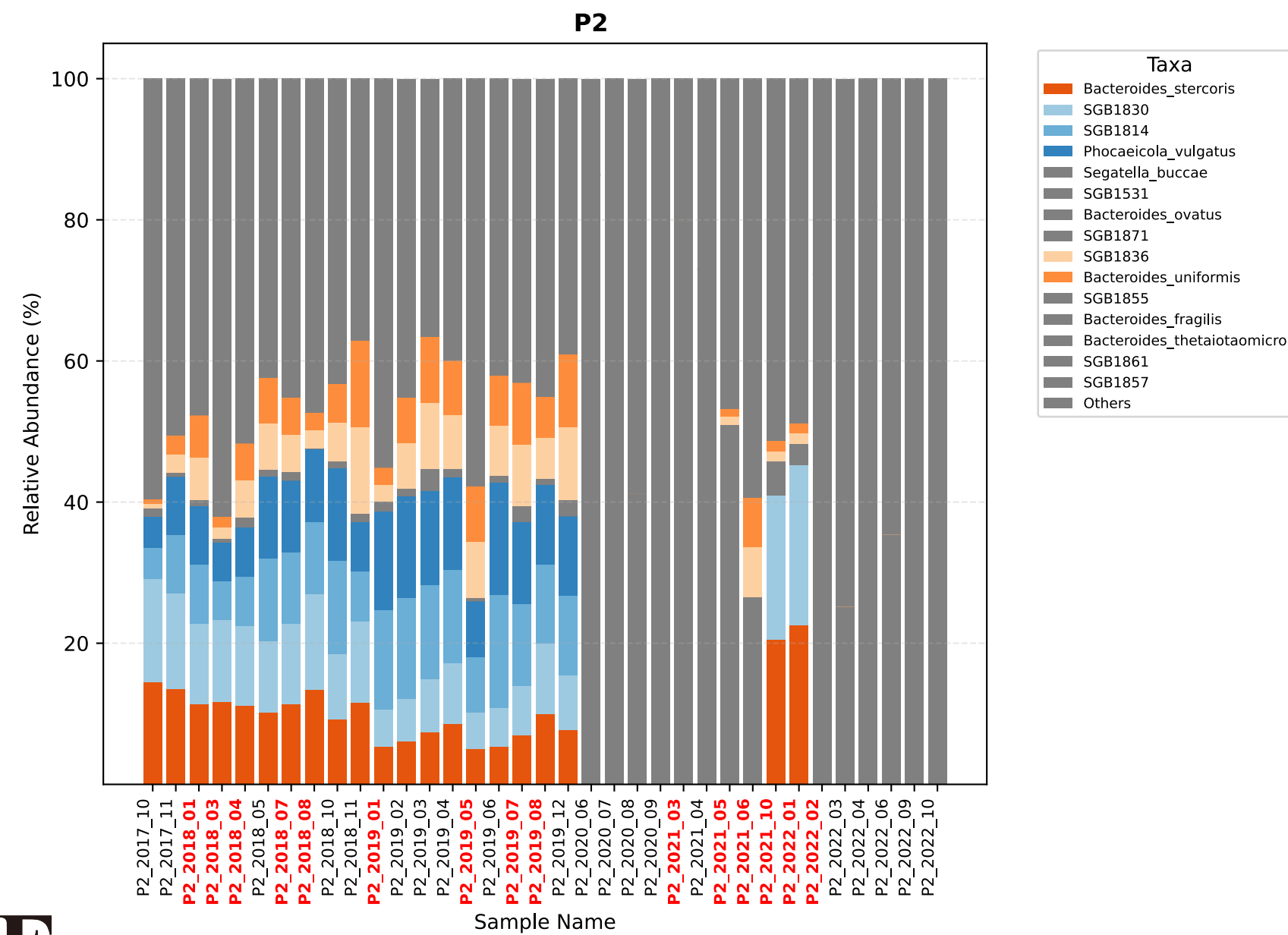

C

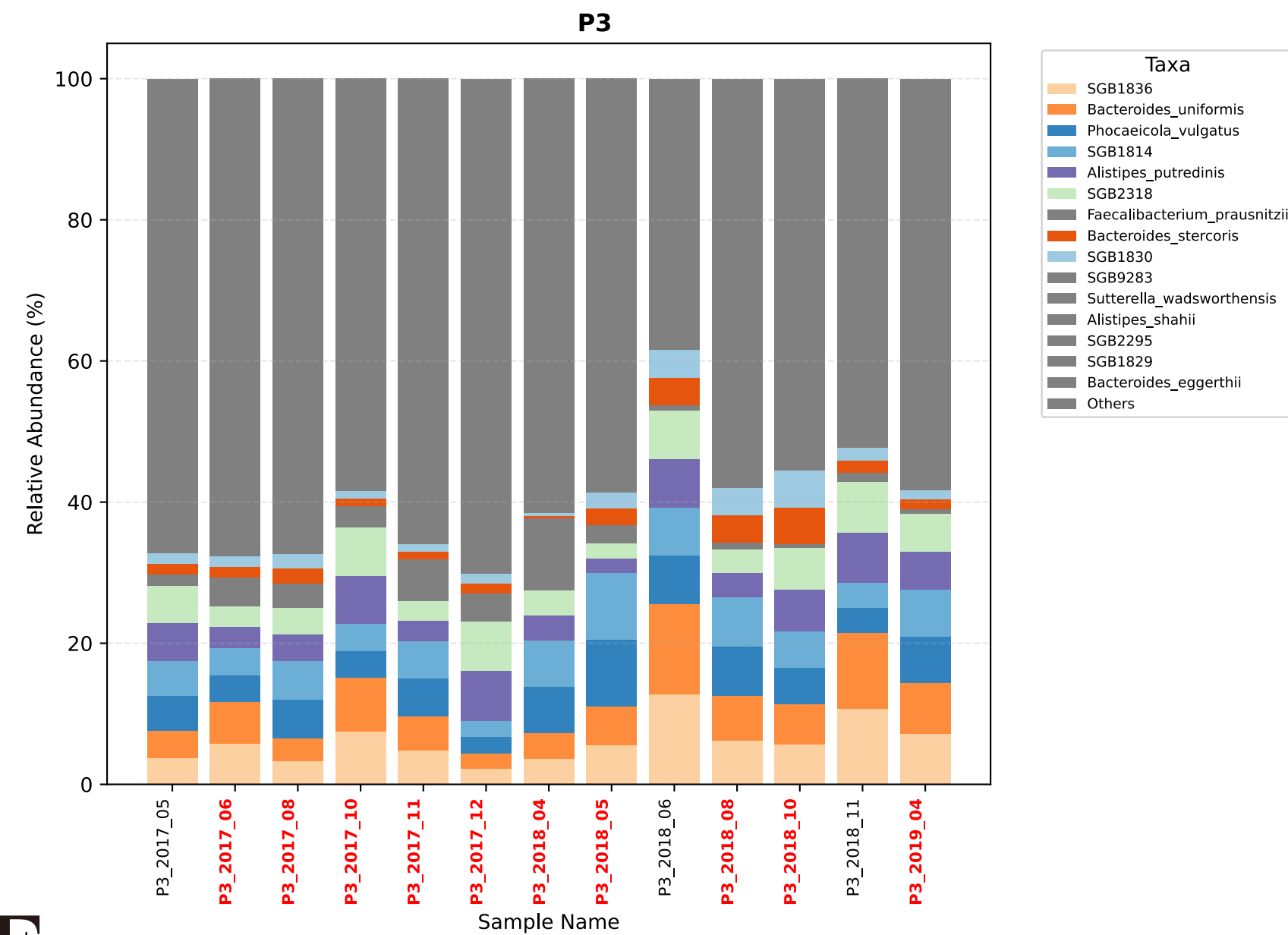

D

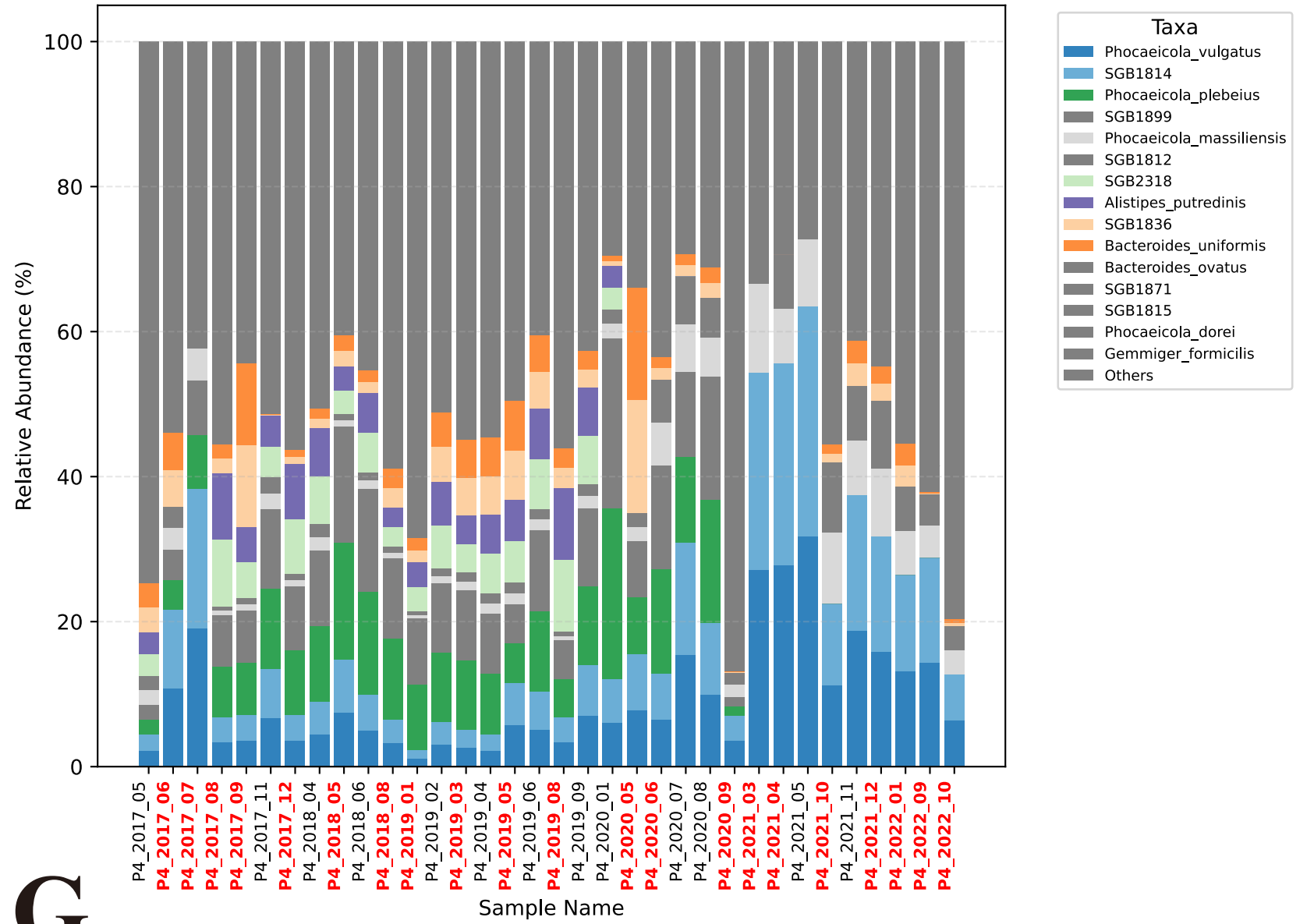

E

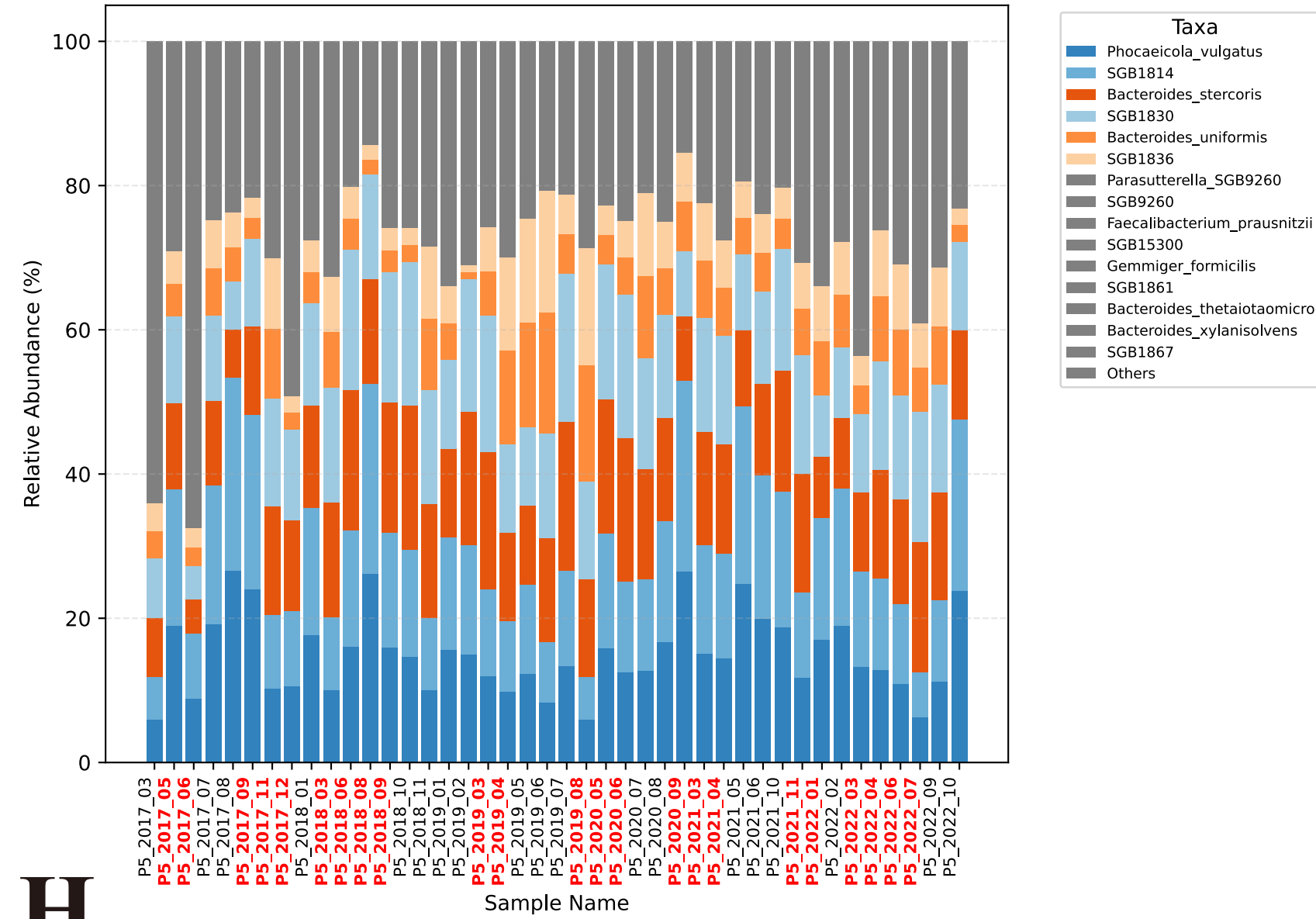

F

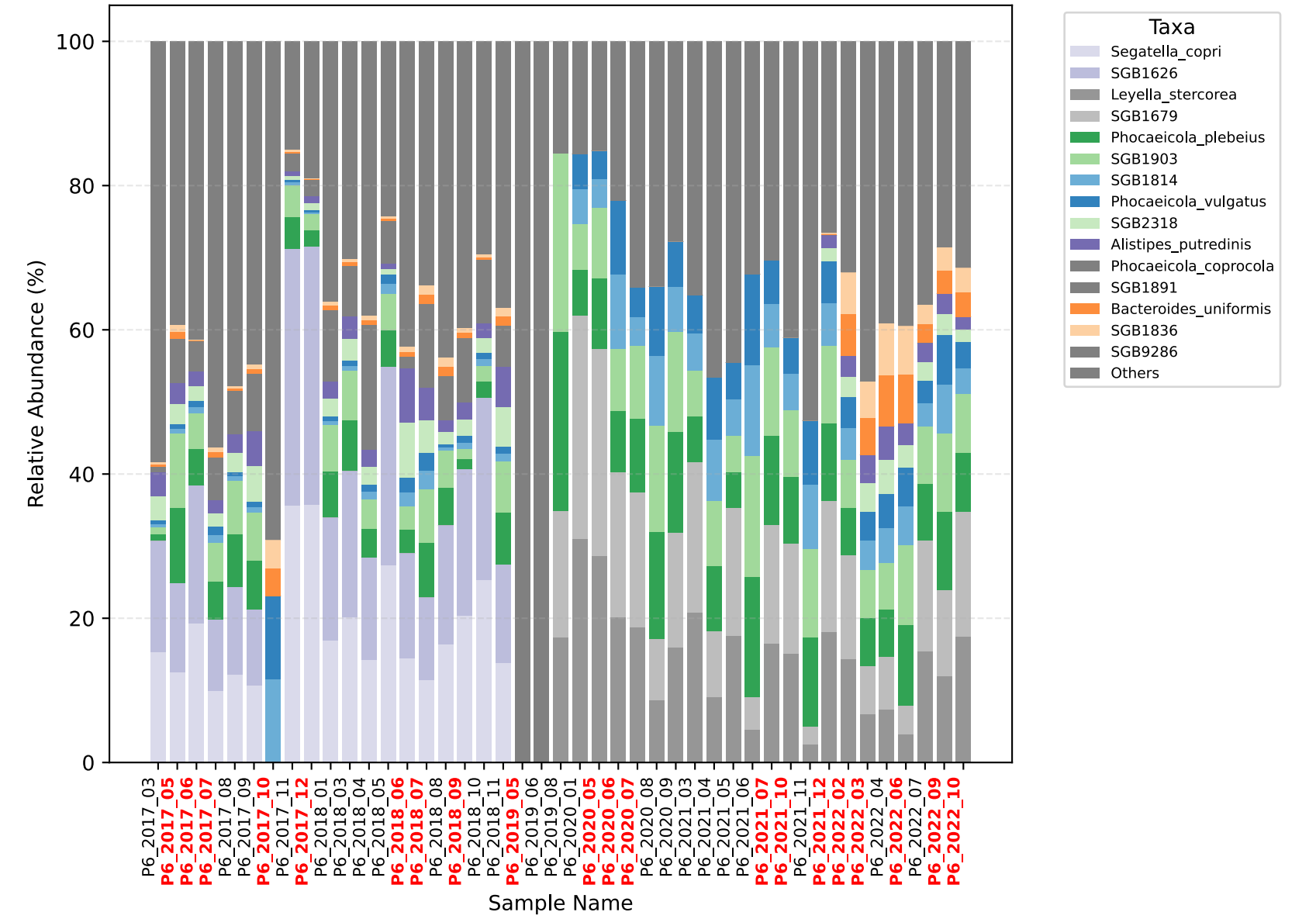

G

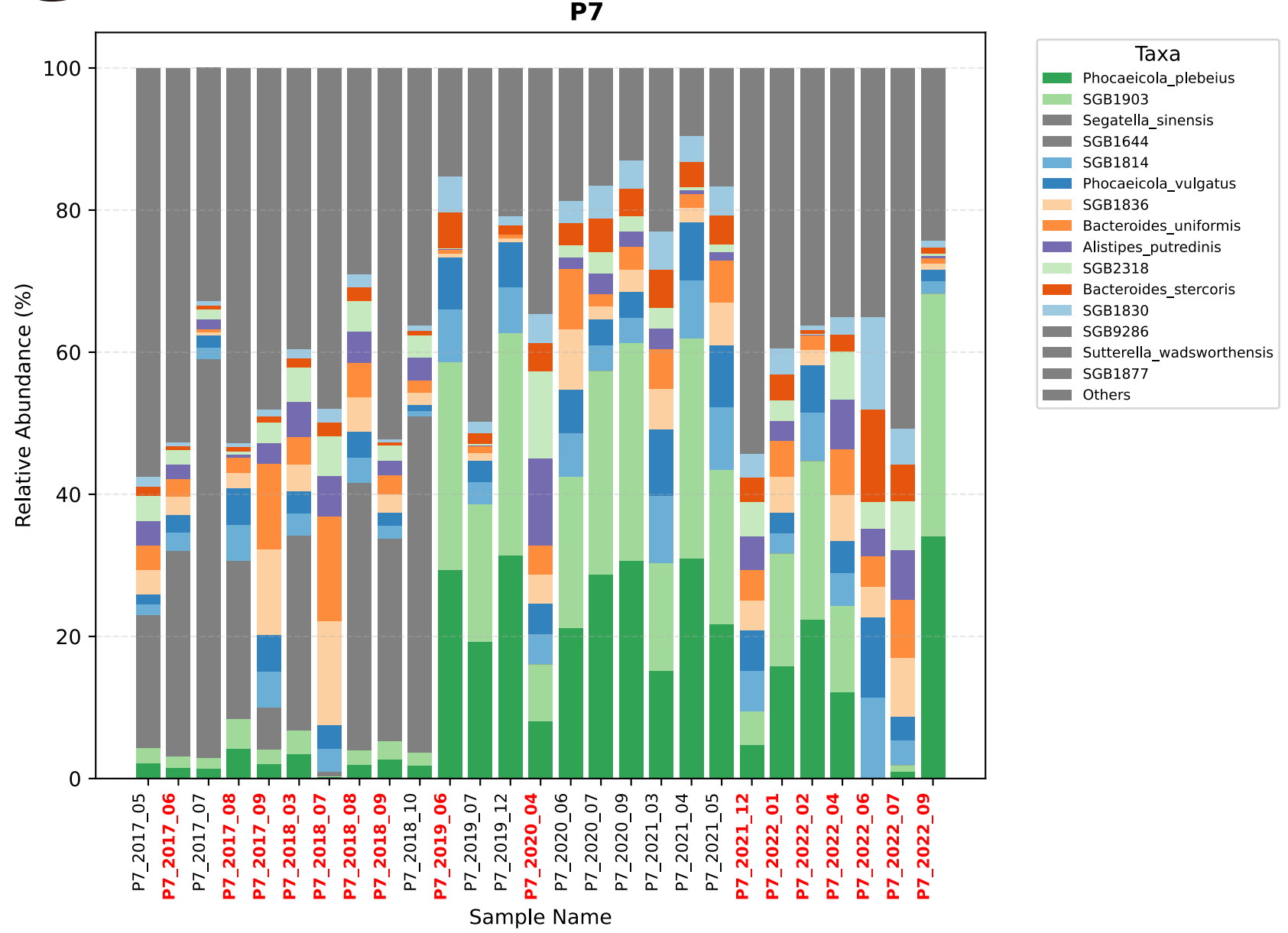

H

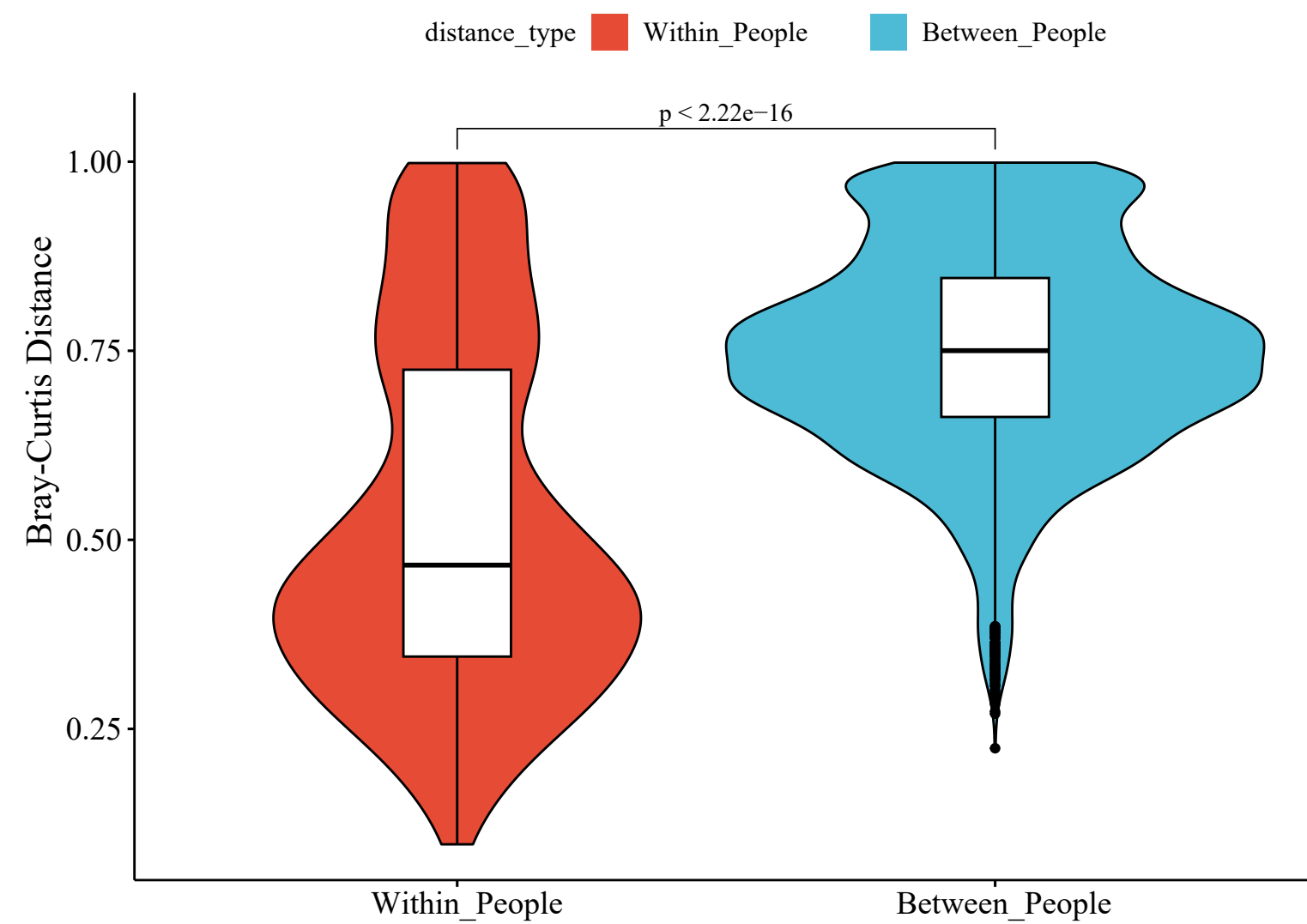

Supplement: Supplementary_material_ycag046 [file supplementary_material_ycag046.zip › SFig1.pdf]

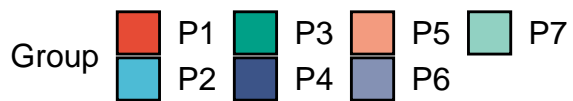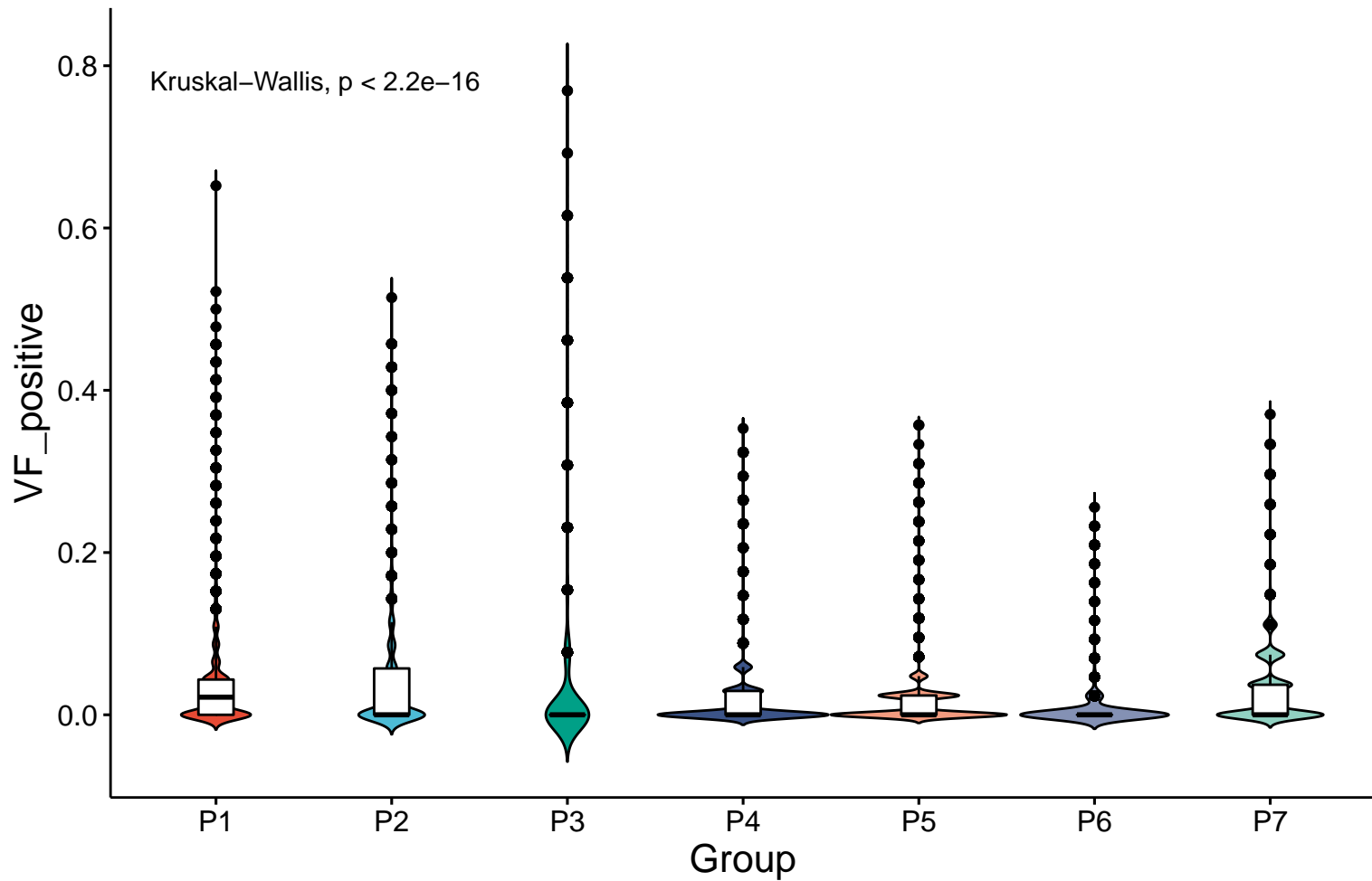

Supplement: Supplementary_material_ycag046 [file supplementary_material_ycag046.zip › SFig2_VF_positive.pdf]

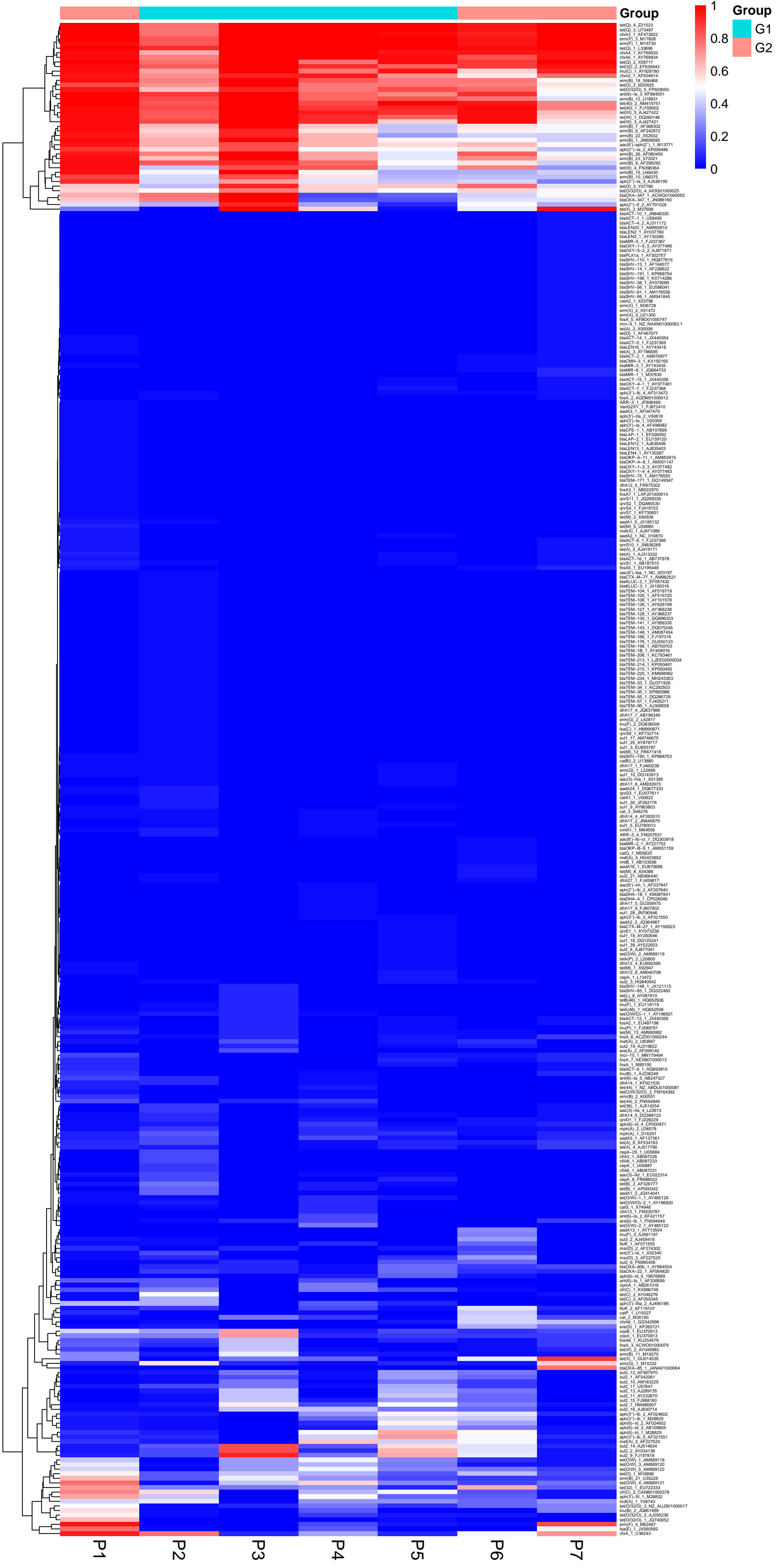

Supplement: Supplementary_material_ycag046 [file supplementary_material_ycag046.zip › SFig3_ARG_heatmap.pdf]

## Correlation: TurnOver Rate vs Positive%

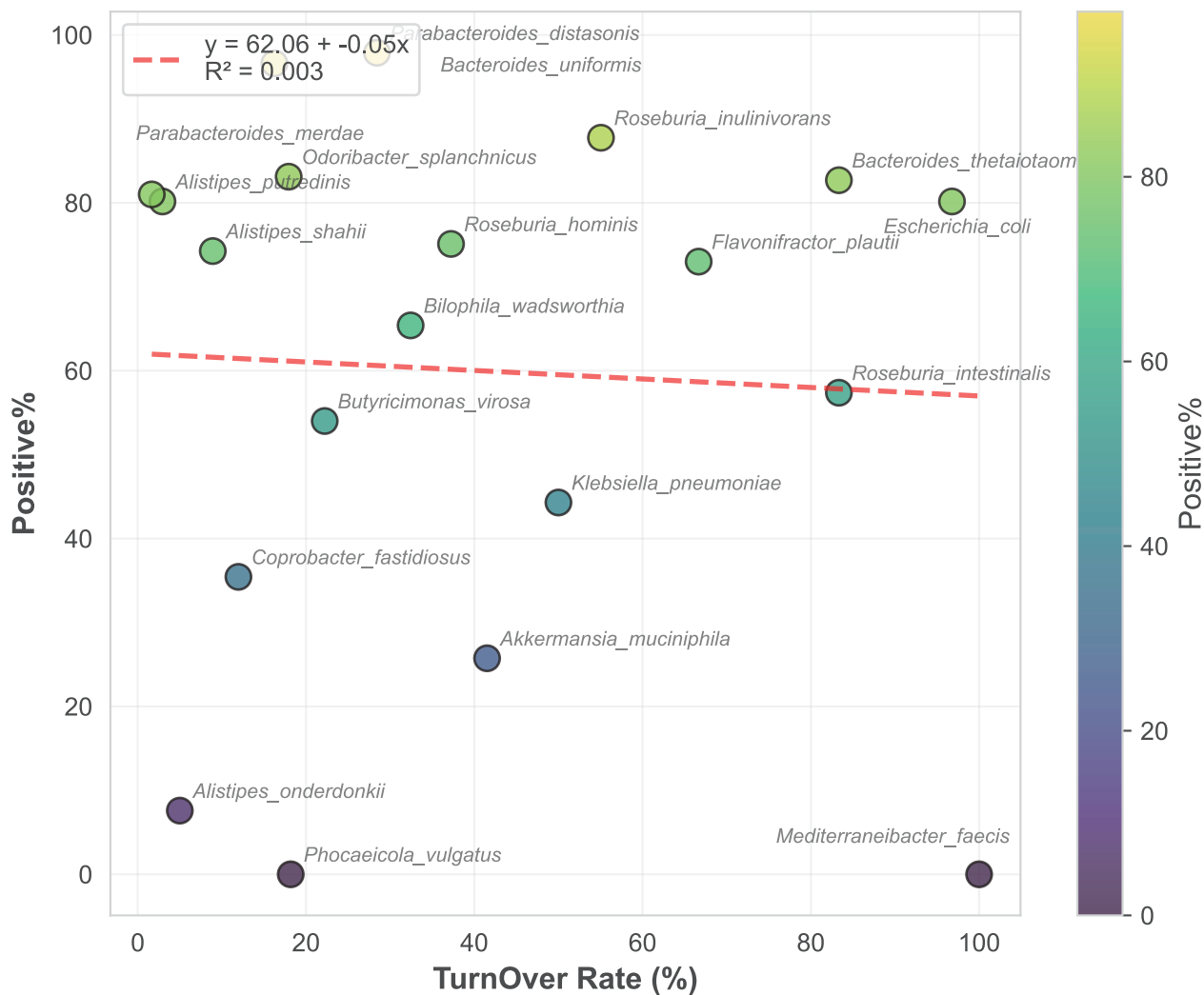

Supplement: Supplementary_material_ycag046 [file supplementary_material_ycag046.zip › SFig4.pdf]
